# WT EGFR and the oncogenic mutant EGFR (L858R) employ distinct mechanisms for export from the endoplasmic reticulum, a process critical for their activation

**DOI:** 10.1016/j.jbc.2025.110312

**Published:** 2025-05-29

**Authors:** Mo Wang, Pik Ki Lau, Yusong Guo

**Affiliations:** 1 Division of Life Science and State Key Laboratory of Molecular Neuroscience, The Hong Kong University of Science and Technology, Hong Kong, China; 2 Hong Kong University of Science and Technology Shenzhen Research Institute, Shenzhen, China

**Keywords:** COPII, ER, Golgi, EGFR, cargo sorting

## Abstract

The epidermal growth factor receptor (EGFR) is critical for cell differentiation, growth, proliferation, and migration. To function, newly synthesized EGFR must be transported from the endoplasmic reticulum (ER) to the cell surface, yet the specific molecular mechanisms mediating this trafficking are not fully understood. We found that the ER export of EGFR is facilitated by a conserved polyarginine (polyR) motif located in the juxtamembrane region of the EGFR cytosolic domain. Mechanistic studies show that this motif interacts directly with the D198 residue on SAR1A, regulating the incorporation of EGFR into COPII vesicles for delivery to the Golgi. A depletion of the polyR motif impairs epidermal growth factor–induced EGFR activation. Interestingly, we found that the ER export of the oncogenic mutant EGFR (L858R) is critical for its activation but does not depend on the D198 residue of SAR1A or the polyR motif of EGFR. Our study elucidates a mechanism regulating ER export of WT EGFR and indicates that the ER exports of the EGFR^L858R^ mutant and WT EGFR are mediated by distinct molecular machineries, essential for their activation.

The epidermal growth factor receptor (EGFR) is a cell surface receptor that binds epidermal growth factor (EGF) and transforming growth factor-α. Binding of these ligands activates EGFR, initiating signaling pathways that promote cell growth and proliferation. Due to its significant role in cancer progression, EGFR is an important target for targeted cancer therapies.

Newly synthesized EGFR follows the conventional steps in the secretory transport pathway to be delivered to the cell surface. After being synthesized from ribosomes, EGFR is first translocated in the membrane of the endoplasmic reticulum (ER). After the folding and modification steps at the ER, EGFR is packaged into transport vesicles that are targeted to the Golgi apparatus en route to the cell surface. The key player that mediates export of cargo proteins out of the ER is the COPII coat. The COPII coat is composed of five cytosolic proteins: the small GTPase SAR1, the SEC23/24 heterodimer, and the SEC13/31 heterodimer (1). Assembly of the COPII coat at the ER is initiated by SAR1. Upon GTP binding, SAR1 recruits the SEC23/24 heterodimer, forming the inner COPII coat. Subsequently, the inner COPII coat recruits the SEC13/31 heterodimer, which is thought to polymerize the coat and drive membrane deformation to generate COPII vesicles (1). In addition to driving vesicle formation, the COPII coat recognizes specific sorting motifs in the cytosolic domain of transmembrane cargo proteins, thereby enriching them into budding vesicles (1).

EGFR undergoes internalization and degradation after treating the cells with EGF. Interestingly, prolonged EGF treatment enhanced the efficiency of delivery of newly synthesized EGFR from the ER to the plasma membrane. Further analyses indicate that ER export of EGFR depends on the COPII subunits, SEC24B, SEC24D, and SEC23B. Coincidently, the expression of these COPII subunits is upregulated upon EGF treatment. This upregulation depends on a transcription factor, RNF11, which appears in the nucleus upon EGF treatment (2). According to these analyses, there appears to be a feed-forward loop operating within the cell that regulates the synthesis and transport of EGFR to optimize its efficiency and maintain the appropriate level of EGFR on the cell surface. Currently, it is unclear whether the COPII subunits SEC24B or SEC24D directly interact with EGFR.

Many oncogenic mutations in EGFR have been identified in cancer cells, with one of the most prevalent being the L858R point mutation in exon 21, occurring in approximately 40% of lung cancer cases (3, 4). This L858R mutation is located in the activation loop of the EGFR kinase domain. Structural analyses have shown that while the WT EGFR kinase domain typically adopts an inactive conformation, the L858R mutation disrupts interactions that maintain this inactive state, effectively keeping the enzyme in a perpetually active state (5). In addition, the L858R mutation enhances EGFR dimerization, which activates the kinase activity (3). These structural changes indicate that the EGFR L858R variant might engage with distinct cellular factors affecting its intracellular movement. It remains uncertain whether the WT and oncogenic mutant forms of EGFR utilize the same ER export mechanisms.

In this study, we utilized the Retention Using Selective Hook (RUSH) transport assay to analyze the ER export of EGFR in a synchronized manner and reconstituted the packaging of EGFR into COPII vesicles *in vitro* to measure cargo capture efficiency. These methods, combined with other experimental approaches, allowed us to identify the ER export motif of EGFR and elucidate the molecular mechanism that enhances the enrichment of WT EGFR into COPII vesicles. Our findings reveal that WT EGFR and the EGFR L858R mutant rely on distinct cellular factors for their delivery from the ER to the Golgi. In addition, we discovered that the activation of both WT and L858R mutant EGFR is contingent upon ER export, highlighting the inhibition of EGFR ER export as a viable strategy for targeting EGFR-related cancers.

## Results

### ER export of EGFR depends on its polyarginine motif

**Figure 1:**
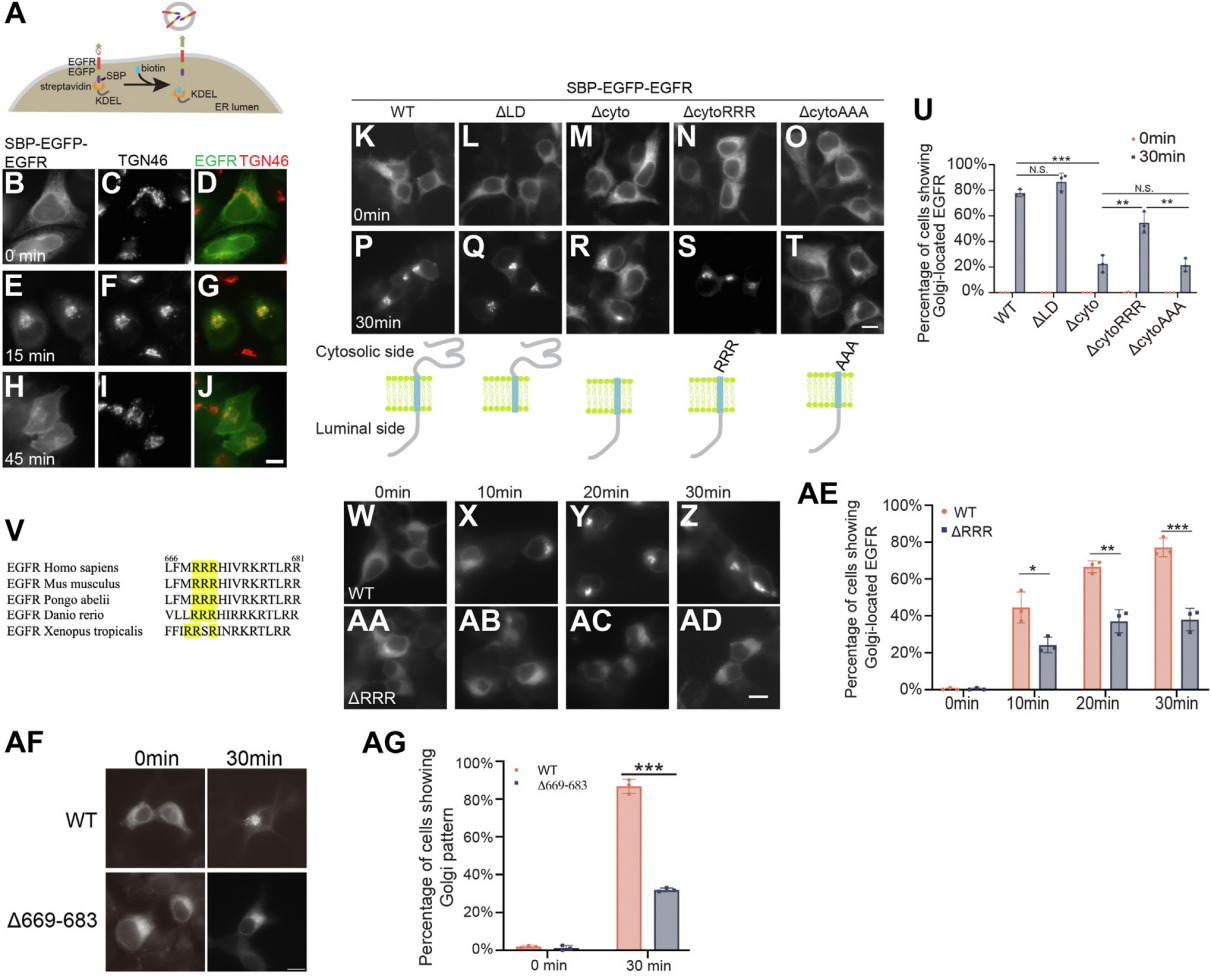
**ER export of EGFR depends on its polyR motif**. *A*, a diagram demonstrating the RUSH transport assay. *B*–*J*, HeLa cells were transfected with plasmids expressing Str-KDEL and SBP–EGFP–EGFR and treated with biotin for the indicated period. The localizations of the indicated proteins was analyzed by fluorescence microscopy. Scale bar represents 10 μm. *K*–*T* and *W*–*AD*, HEK293T cells were transfected with plasmids encoding the RUSH construct of WT EGFR or mutant versions of EGFR. The localizations of these EGFR constructs were analyzed after incubating with biotin for the indicated period. Scale bar represents 10 μm. *U* and *AE*, percentages of cells showing juxtanuclear-accumulated EGFR were quantified (n = 3, mean ± SD, over 100 cells were quantified in each experiment group). *V*, sequence alignment of the juxtamembrane region of the cytosolic domain EGFR from different species. *AF*, HEK293T cells were transfected with plasmids encoding the RUSH construct of WT EGFR or RUSH-EGFR^Δ669–683^. The localizations of these EGFR constructs were analyzed after incubating with biotin for the indicated period. Scale bar represents 10 μm. *AG*, percentages of cells showing juxtanuclear-accumulated EGFR were quantified (n = 3, mean ± SD, over 100 cells were quantified in each experiment group). ∗*p* < 0.05; ∗∗*p* < 0.01; and ∗∗∗*p* < 0.001. EGFP, enhanced GFP; EGFR, epidermal growth factor receptor; ER, endoplasmic reticulum; HEK293T, human embryonic kidney 293T cell line; LD, luminal domain; NS, not significant; polyR, polyarginine motif; RUSH, Retention Using Selective Hook; SBP, streptavidin-binding peptide.

We performed the RUSH transport assay (6) to study delivery of newly synthesized EGFR from the ER to the plasma membrane. In the RUSH assay, EGFR was tagged with enhanced GFP (EGFP) and streptavidin-binding peptide (SBP) (SBP–EGFP–EGFR). The plasmids expressing SBP–EGFP–EGFR also encode streptavidin conjugated with an ER retention motif KDEL (Str-KDEL). SBP–EGFP–EGFR was retained at the ER because of the binding between SBP and Str-KDEL (Fig. 1, *A*–*D*). Upon biotin treatment, SBP–EGFP–EGFR was released from the ER (Fig. 1*A*). Fifteen minutes after biotin treatment, SBP–EGFP–EGFR was accumulated at the juxtanuclear Golgi area (Fig. 1, *E*–*G*). Forty-five minutes after biotin treatment, the signal intensity of SBP–EGFP–EGFR at the Golgi area decreased gradually, which was followed by an increase of its abundance on the plasma membrane (Fig. 1, *H*–*J*). This RUSH assay suggests that the surface delivery of EGFR follows the conventional secretory pathway, which is consistent with previous reports (2, 7).

EGFR is a type I single-pass transmembrane protein. To investigate the mechanism of EGFR export out of the ER, we generated several truncated EGFR constructs in the RUSH system. These constructs include WT EGFR (SBP–EGFP–EGFR^WT^), EGFR deleted of its luminal domain (SBP–EGFP–EGFR^ΔLD^), and EGFR deleted of its cytosolic domain (SBP–EGFP–EGFR^Δcyto^). All these constructs were retained at the ER in the absence of biotin (Fig. 1, *K*–*M*). After biotin treatment for 30 min, the WT EGFR was efficiently delivered from the ER to the Golgi (Fig. 1, *P* and *U*). Deleting the luminal domain did not affect the efficiency of ER-to-Golgi delivery (Fig. 1, *Q* and *U*), whereas deleting the cytosolic domain caused a strong retention of EGFR at the ER in the majority of cells after biotin treatment (Fig. 1, *R* and *U*). A conserved triple arginine motif, consisting of amino acids 669 to 671 and referred to as polyarginine motif (polyR motif) was identified at the juxtamembrane region on EGFR cytoplasmic domain (Fig. 1*V*). The polyR motif is present as an ER export motif in a planar cell polarity protein, Frizzled6 and many glycolipid glycosyltransferases (8, 9). Adding back the polyR motif to the Δcyto construct of EGFR (SBP–EGFP–EGFR^ΔcytoRRR^) significantly increased the efficiency of ER-to-Golgi trafficking (Fig. 1, *S* and *U*), whereas adding back the triple alanine instead of the triple arginine to the Δcyto construct (SBP–EGFP–EGFR^ΔcytoAAA^) did not enhance the efficiency (Fig. 1, *T*–*U*). Interestingly, deleting the polyR motif (SBP–EGFP–EGFR^ΔRRR^) significantly reduced the efficiency of ER-to-Golgi trafficking of SBP–EGFP–EGFR (Fig. 1, *W*–*AE*). These results indicate that the polyR motif is important for ER export of EGFR.

The ΔRRR construct still has RKR and RR motifs intact. To further investigate the role of the remaining RKR and RR motifs, we generated a deletion construct (Δ669–683) that removes the entire polybasic region (RRRHIVRKRTLRRLL) and assessed its ER export using the RUSH assay. As shown in Figure 1*AF*, RUSH-EGFR^Δ669-683^ exhibited strong ER localization after 30 min of biotin treatment. Quantification revealed that only ∼30% of cells expressing this construct showed a Golgi localization pattern (Fig. 1*AG*), which is comparable to the retention observed with RUSH-EGFR^ΔRRR^ (Fig. 1*AE*). These results demonstrate that the polyR motif (amino acids 669–671) plays the dominant role in EGFR ER export, whereas the downstream RKR and RR motifs contribute minimally, as their removal did not significantly enhance ER retention.

### The polyR motif is required for packaging of EGFR into COPII vesicles

**Figure 2:**
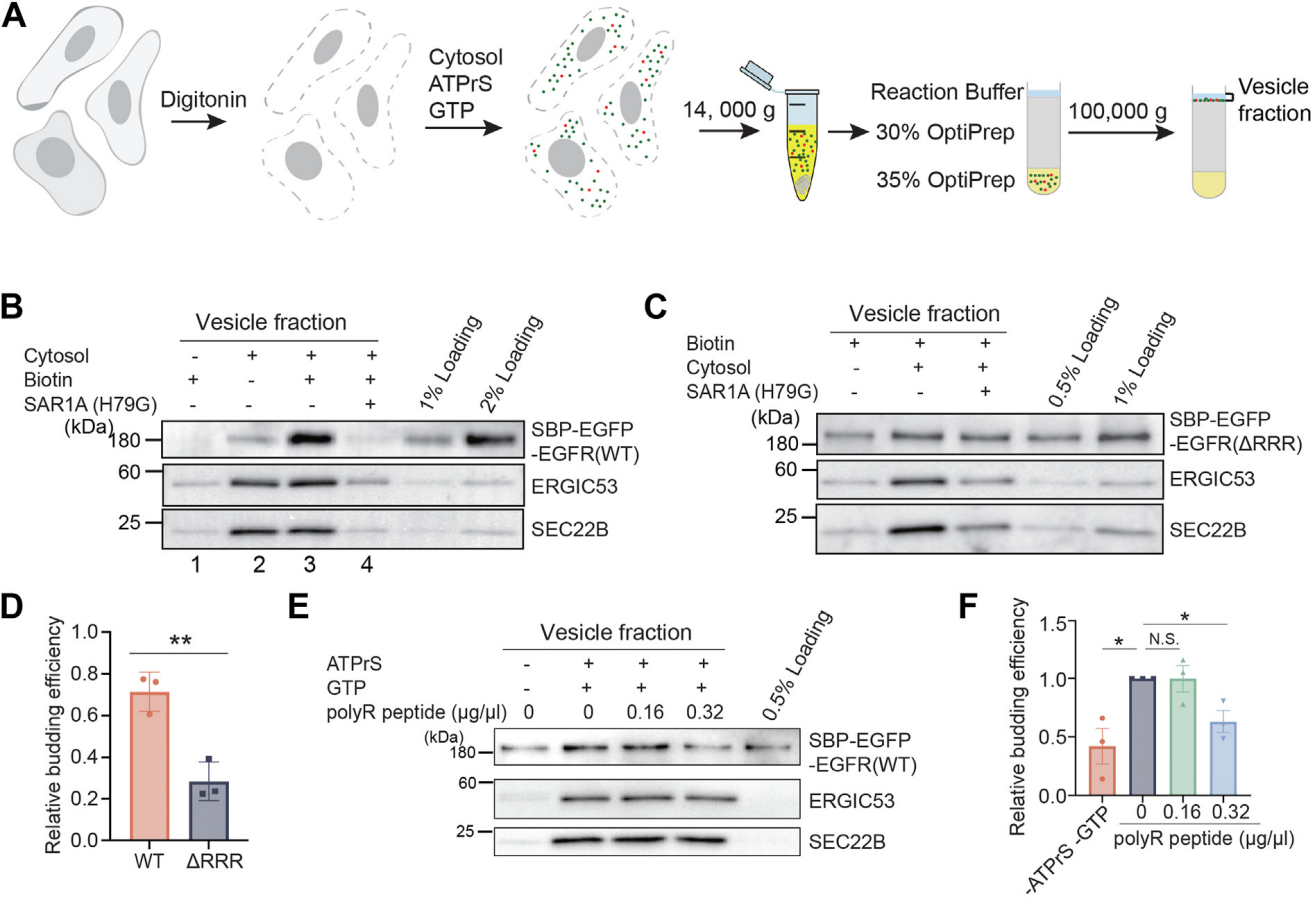
**The polyR motif is required for packaging of EGFR into COPII-coated vesicles**. *A*, a diagram demonstrating the procedures of the vesicle formation assay. *B* and *C*, the vesicle formation assay was performed using HEK293T cells expressing SBP–EGFP–EGFR^WT^ or SBP–EGFP–EGFR^ΔRRR^. Vesicle fractions were analyzed by immunoblot. *D*, quantification of the budding efficiency of EGFR^WT^ and EGFR^ΔRRR^. The abundance of EGFR in “+biotin + cytosol-SAR1(H79G)” was normalized with corresponding 1% loading. ∗∗*p* < 0.01. *E*, the vesicle formation assay was performed using HEK293T cells expressing SBP–EGFP–EGFR^WT^ in the presence of the polyR peptides under different concentrations. Vesicle fractions were then analyzed by immunoblot. *F*, the relative budding efficiency of SBP–EGFP–EGFR^WT^ was quantified (n = 3, mean ± SD). The quantification was normalized to the abundance of EGFR in the vesicle fraction from the experimental group performed without peptide. ∗*p* < 0.05; NS, not significant. EGFP, enhanced GFP; EGFR, epidermal growth factor receptor; HEK293T, human embryonic kidney 293T cell line; polyR, polyarginine motif.

Next, we sought to test whether the polyR motif is essential for enrichment of EGFR into COPII vesicles. The *in vitro* vesicle formation assay was utilized to reconstitute the release of EGFR into vesicles at the ER. This assay has been well established to quantify the efficiency of cargo capture into COPII vesicles (10, 11) (Fig. 2*A*). Human embryonic kidney 293T (HEK293T) cells overexpressing the RUSH constructs of EGFR were treated with digitonin to disrupt the plasma membrane. Semi-intact cells were then incubated with an ATP regeneration system, GTP and rat liver cytosol in the presence or the absence of biotin. After incubation at 32°C for 1 h, the cell debris and large membranes were removed by medium-speed centrifugation. The released vesicles were isolated through a density gradient flotation assay. The top fraction after floatation was concentrated by high-speed centrifugation and analyzed by Western blot.

The RUSH construct of WT EGFR (SBP–EGFP–EGFR^WT^) showed cytosol-dependent packaging into vesicles (Fig. 2*B*, compare lanes 1 and 3). The efficiency of packaging was greatly enhanced in the presence of biotin (Fig. 2*B*, compare lanes 2 and 3). Addition of purified His-tagged GTPase-defective mutant form of SAR1A, SAR1A(H79G), strongly inhibited vesicular release of EGFR and the standard COPII cargo proteins, SEC22B and ERGIC53 (Fig. 2*B*, compare lanes 3 and 4). This result indicates that this assay recapitulates the requirements of packaging of EGFR into vesicles from the ER in intact cells. The budding efficiency of SBP–EGFP–EGFR^ΔRRR^, quantified as the fraction of EGFR packaged into transport vesicles from semi-intact cells in the presence of biotin and cytosol, was significantly reduced compared with SBP–EGFP–EGFR^WT^ (Fig. 2, *B*–*D*). In addition, SAR1A(H79G) did not cause an obvious reduction of the budding efficiency of SBP–EGFP–EGFR^ΔRRR^ (Fig. 2*C*). Moreover, addition of polyR peptide corresponding to the EGFR polyR motif significantly reduced the budding efficiency of SBP–EGFP–EGFR^WT^ but not SEC22B at a concentration-dependent manner (Fig. 2, *E* and *F*). These results indicate that the polyR motif is essential for packaging of EGFR into COPII vesicles.

### The D198 residue on SAR1A directly interacts with the polyR motif on EGFR, and this residue is important for ER export of EGFR

**Figure 3:**
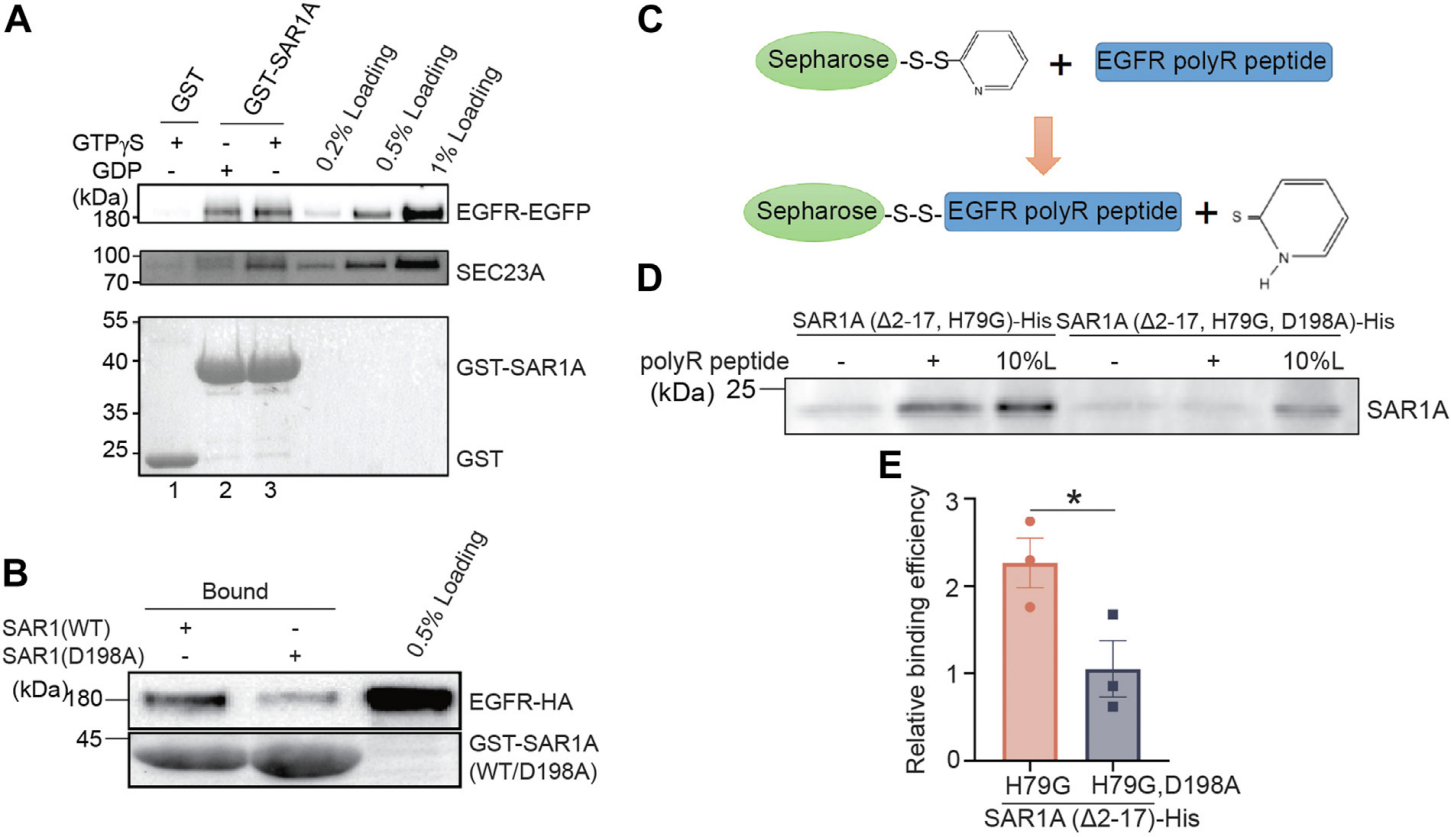
**The polyR motif in EGFR directly interacts with the D198 residue on human SAR1A**. *A*, COS7 cells were transfected with EGFR–EGFP and harvested for GST pull-down assay with purified GST or GST-SAR1A in the presence of GDP or GTPγS. The bound proteins and cell lysates were then analyzed by immunoblot. *B*, HEK293T cells were transfected with EGFR-HA, and cells were harvested for GST pull down assay with GST-tagged WT SAR1A or GST-SAR1A^D198A^. The bound proteins and cell lysates were then analyzed by immunoblot. *C*, a diagram demonstrating the peptide-binding assay. *D*, polyR peptides were covalently linked to thiopyridone-Sepharose 6B, incubated with purified His-tagged SAR1A^Δ2–17, H79G^, or His-tagged SAR1A^Δ2–17, H79G, D198A^. After incubation, the bound proteins were analyzed by immunoblot. *E*, quantifications of the relative abundances of SAR1A^Δ2–17, H79G^ and SAR1A^Δ2–17, H79G, D198A^ bound to the polyR peptides. The relative abundances of bound proteins were normalized to that in the experimental group without the polyR peptides (n = 3, mean ± SD). ∗*p* < 0.05. EGFP, enhanced GFP; EGFR, epidermal growth factor receptor; GST, glutathione-*S*-transferase; HEK293T, human embryonic kidney 293T cell line; polyR, polyarginine motif.

We hypothesized that the polyR motif on EGFR functions as the recognition site for EGFR recruitment into COPII vesicles. We tested the interaction between COPII coat subunit SAR1A and EGFR through glutathione-*S*-transferase (GST) pull-down assay. GST and GST-tagged SAR1A were purified and incubated with cell lysate from COS7 cells overexpressing EGFR–EGFP, in the presence of GDP or GTPγS. The bound proteins were then analyzed by immunoblotting. We found that EGFR–EGFP in the cell lysate showed slightly higher abundance of binding to GST-SAR1A in the presence of GTPγS compared to the group containing GDP. However, no binding to GST was detected (Fig. 3*A*, lanes 1–3). SEC23A, a COPII component known to interact with GTP-bound SAR1A, served as a positive control (Fig. 3*A*). These results suggest that EGFR interacts with SAR1A.

The polyR motif in glycolipid glycosyltransferases has been shown to directly bind to the aspartate198 residue on SAR1A to facilitate the enrichment of these enzymes at the ER exit sites (9). To verify whether the D198 residue on SAR1A is also important for SAR1A to bind to EGFR, we generated a GST-tagged SAR1A mutant, GST-SAR1A^D198A^, in which the D198 residue was mutated to alanine. GST pull-down analysis indicates that D198A substitution greatly reduced the abundance of EGFR that bound to GST-SAR1A (Fig. 3*B*).

We performed a peptide binding assay to test whether EGFR polyR motif directly binds to SAR1A. Synthetic polyR peptide (sequence RRRHIVRKRTLRR, amino acid number: 669-681) was covalently conjugated to thiopyridone-Sepharose 6B beads (Fig. 3*C*). The beads were incubated with purified His-tagged SAR1A(H79G) deleted of its N-terminal helix (SAR1A^Δ2–17, H79G^-His) and analyzed by immunoblotting. Intriguingly, we found that purified SAR1A^Δ2–17, H79G^-His directly interacted with the synthetic polyR peptides (Fig. 3*D*). On the contrary, the D198A substitution significantly reduced the abundance of SAR1A that bound to the polyR peptides (Fig. 3, *D* and *E*), indicating that the polyR motif on EGFR directly interacts with the D198 residue on SAR1A.

**Figure 4:**
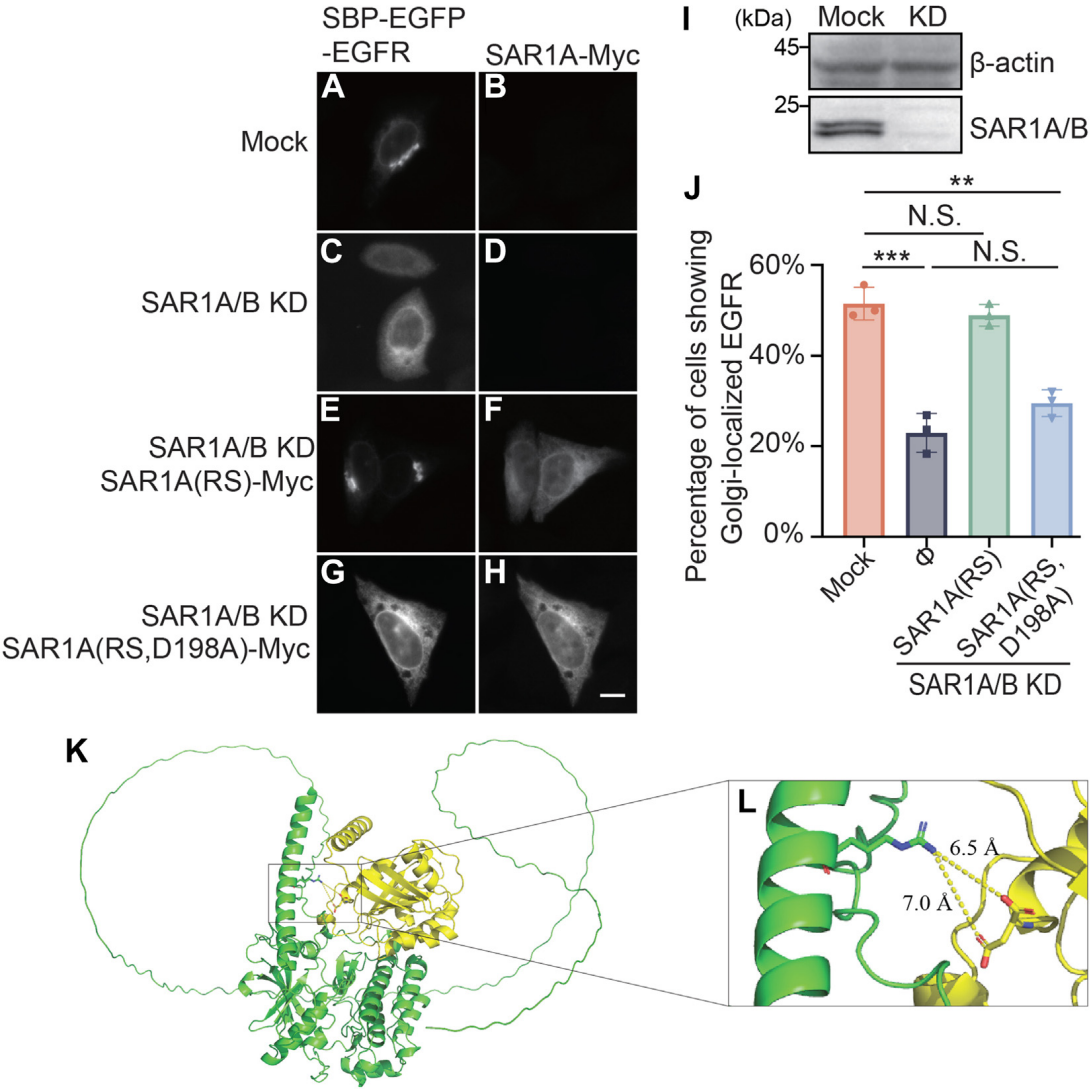
**The D198 residue of SAR1 is required for ER export of EGFR**. *A*–*H*, the RUSH transport assay was performed in HeLa cells transfected with control siRNA (*A* and *B*) or cotransfected with siRNAs against SAR1A and SAR1B. The localizations of SBP–EGFP–EGFR^WT^ were analyzed in cells incubated with biotin for 15 min. Scale bar represents 10 μm. *I*, lysates from cells transfected with control siRNA or siRNAs against SAR1A and SAR1B were analyzed by immunoblot. *J*, the percentage of cells showing juxtanuclear-localized EGFR was quantified (n = 3, mean ± SD, over 100 cells were quantified in each experiment group). ∗∗*p* < 0.01; ∗∗∗*p* < 0.001; NS, not significant. *K*, AlphaFold multimer prediction of EGFR-SAR1A protein–protein interface. (EGFR: *green*; SAR1A: *yellow*). Enlarged binding interface between R669 and D198 showed in *L*. EGFP, enhanced GFP; EGFR, epidermal growth factor receptor; ER, endoplasmic reticulum; RUSH, Retention Using Selective Hook.

We then performed a knockdown and rescue experiment to test whether the D198 residue on SAR1A/B is important for ER export of EGFR. We found that knockdown of endogenous SAR1A and SAR1B caused defects in ER-to-Golgi transport of SBP–EGFP–EGFR^WT^ in the RUSH system (Fig. 4, *A*–*D*, *I*–*J*). This defect was rescued by expressing siRNA-resistant SAR1A construct bearing nonsense mutations in the siRNA target area (Fig. 4, *E*, *F* and *J*). In contrast, expression of an siRNA-resistant SAR1A D198A mutant in SAR1-knockdown cells failed to rescue (Fig. 4, *G*, *H* and *J*). We conducted AlphaFold predictions using both full-length EGFR and SAR1A (Fig. 4*K*). The model revealed a potential interaction between the R669 residue of EGFR and the D198 residue of SAR1A, with a predicted distance of 6.5 Å between these residues (Fig. 4*L*). This relatively large separation suggests the interaction is likely weak, consistent with typical cargo–adaptor interactions. These data indicate that the D198 residue of SAR1 is required for ER export of EGFR.

### The export of EGFR from the ER is crucial for its activation

**Figure 5:**
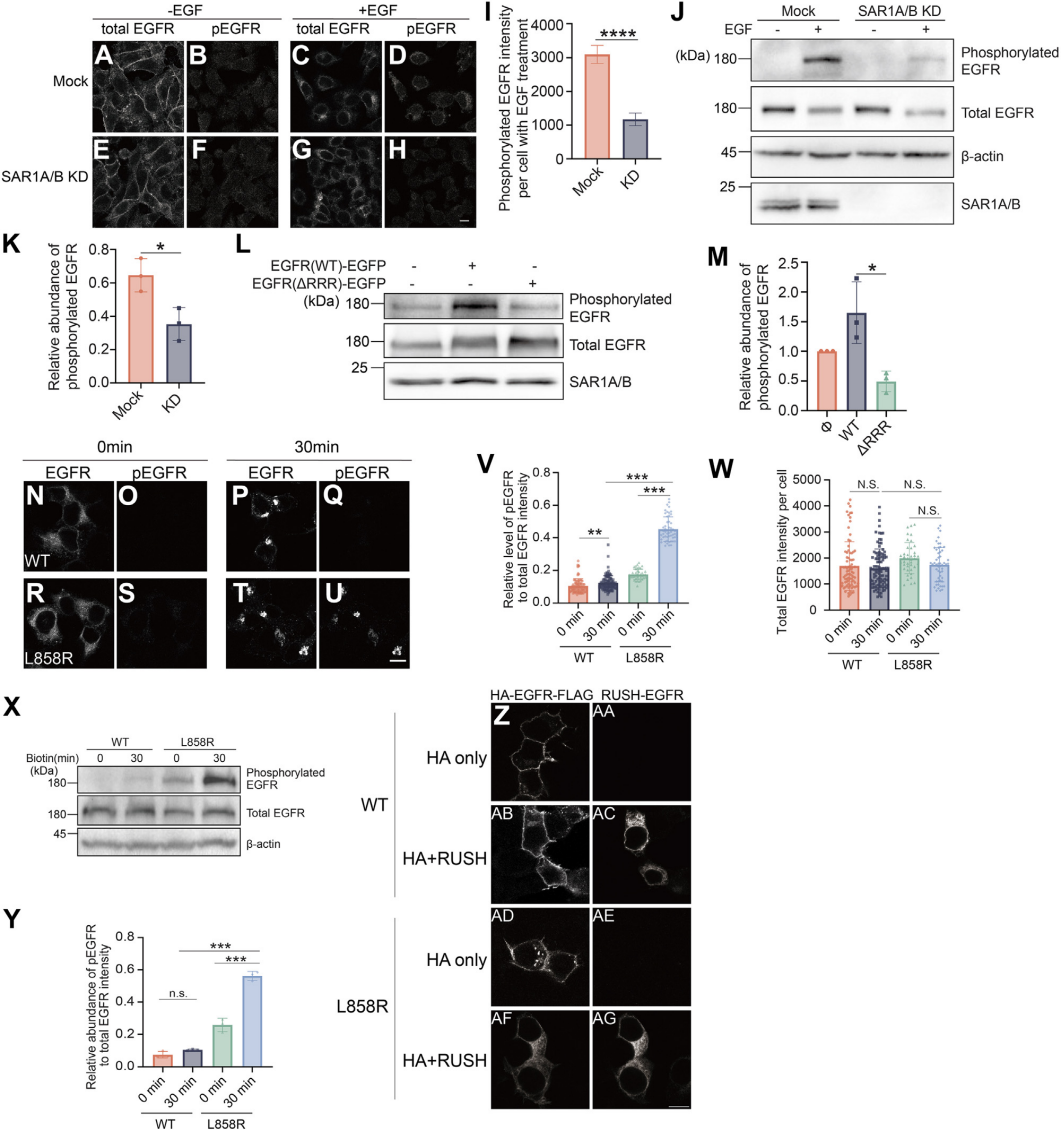
**The export of EGFR from the ER is crucial for its activation**. *A*–*H*, HeLa cells were transfected with control siRNA or cotransfected with siRNAs against SAR1A and SAR1B. On day 2 after transfection, cells were incubated with or without 10 ng/ml EGF overnight, and the localizations of the indicated proteins were analyzed by immunofluorescence. Scale bar represents 10 μm. *I*, the intensities of pEGFR signal in cells treated with EGF were quantified. (n > 100 in each experimental group, mean ± SEM) ∗∗∗∗*p* < 0.0001. *J*, HeLa cells were transfected with control siRNA or cotransfected with siRNAs against SAR1A and SAR1B. On day 2 after knockdown, cells were incubated with or without 10 ng/ml EGF overnight. The cell lysates were analyzed by immunoblot. *K*, the levels of pEGFR from mock cells and SAR1A/B KD cells treated with EGF were quantified (n = 3, mean ± SD). In each experimental group, the levels of pEGFR were normalized to the abundance of total EGFR. The sum of the normalized value of the two experimental groups was then normalized to 1. ∗*p* < 0.05. *L*, HeLa cells were transfected with or without WT EGFR–EGFP or EGFR^ΔRRR^–EGFP. Day 1 after transfection, cells were incubated with 10 ng/ml EGF, and the cell lysates were analyzed by immunoblotting with anti-EGFR, anti-pEGFR, and anti-SAR1A/B antibodies. *M*, the levels of pEGFR were quantified (n = 3, mean ± SD). In each experimental group, the level of pEGFR was first normalized to the level of total EGFR. Then the value was normalized to the no-transfection group. ∗*p* < 0.05. *N*–*U*, the RUSH transport assay was performed in HEK293T cells transfected with the indicated RUSH constructs of EGFR^WT^ or EGFR^L858R^. On day 1 after transfection, cells were incubated with biotin and cycloheximide for the indicated time. The levels of total EGFR and phosphorylated EGFR were analyzed by immunofluorescence. Scale bar represents 10 μm. *V* and *W*, the ratio of pEGFR intensity to total EGFR intensity per cell (*V*) and the intensity of total EGFR per cell (*W*) were quantified. (n > 40 in each experimental group, mean ± SD). ∗∗*p* < 0.01; ∗∗∗*p* < 0.001; NS, not significant. *X*, RUSH transport assay was performed in HEK293T cells expressing the indicated RUSH constructs of EGFR^WT^ or EGFR^L858R^. On day 1 after transfection, cells were incubated with biotin and cycloheximide for the indicated time. The cell lysates were analyzed by immunoblotting with indicated antibodies. *Y*, the ratio of the abundance of pEGFR to total abundance of EGFR was quantified (n = 3, mean ± SD). In each experiment, the sum of phosphorylated EGFR ratio in four groups was normalized to 1. ∗∗*p* < 0.01; ∗∗∗*p* < 0.001; NS, not significant. *Z*-*AG*, the localization of the indicated construct of WT or L858R EGFR was analyzed in HEK293T cells. Scale bar represents 10 μm. EGF, epidermal growth factor; EGFP, enhanced GFP; EGFR, epidermal growth factor receptor; ER, endoplasmic reticulum; HEK293T, human embryonic kidney 293T cell line; pEGFR, phosphorylated EGFR; RUSH, Retention Using Selective Hook.

Treating HeLa cells with EGF induces endocytosis and phosphorylation of EGFR (Fig. 5, *A*–*D*), which activates downstream signaling pathways. Newly synthesized WT EGFR must be transported from the ER to the cell surface to fulfill its cellular roles. We hypothesized that inhibiting the ER export of WT EGFR would prevent EGF-induced activation of the receptor. Supporting our hypothesis, a double knockdown of SAR1A and SAR1B impaired the EGF-induced phosphorylation of EGFR in HeLa cells, as determined by immunofluorescence (Fig. 5, *E*–*H* and *I*) and immunoblot analyses (Fig. 5, *J* and *K*). Overexpression of WT EGFR–EGFP significantly increased the abundance of EGF-induced phosphorylation of EGFR, but overexpression of EGFR–EGFP deleted of the polyR motif (EGFR^ΔRRR^–EGFP) did not (Fig. 5, *L*–*M*). Together, these analyses indicate that the export of EGFR from the ER is important for EGF-induced EGFR phosphorylation.

Mutations in the kinase domain of EGFR, such as the commonly occurring L858R mutation found in exon 21, leads to ligand-independent phosphorylation and the constitutive activation of EGFR signaling. The L858R mutation is present in 41% of non–small cell lung cancer patients. Is ER export of EGFR^L858R^ essential for the activation of this oncogenic mutant? To explore this question, we utilized the RUSH system to compare the phosphorylation levels of WT EGFR (EGFR^WT^) and the L858R mutant (EGFR^L858R^) within the ER. The RUSH construct of the WT EGFR (SBP–EGFP–EGFR^WT^) displayed weak phosphorylation signals when accumulated in the ER (Fig. 5, *N*–*O* and *V*). Intriguingly, ER-accumulated SBP–EGFP–EGFR^L858R^ also showed a weak phosphorylation signal (Fig. 5, *R*, *S* and *V*). Subsequently, we triggered the release of these proteins to the Golgi by treating cells with biotin for 30 min. While the relocation of SBP–EGFP–EGFR^WT^ to the Golgi did not enhance its phosphorylation (Fig. 5, *N*–*Q* and *V*), the Golgi-localized SBP–EGFP–EGFR^L858R^ showed a significant increase in phosphorylation compared with when it was ER localized (Fig. 5, *R*–*V*). This was not because of differential expression levels, as both variants maintained comparable expression after 0 and 30 min of biotin treatment (Fig. 5*W*). We further confirmed these findings with an immunoblot assay. In line with the immunostaining results, both EGFR^WT^ and EGFR^L858R^ exhibited weak phosphorylation signals in the absence of biotin treatment. However, upon biotin release for 30 min, EGFR^L858R^ displayed strong phosphorylation, whereas EGFR^WT^ did not (Fig. 5, *X*–*Y*). These findings indicate the importance of ER export in the activation of the oncogenic EGFR^L858R^ mutant.

To test whether ER-retained EGFR^L858R^ is still dimeric, we examined the localization patterns of HA-EGFR-FLAG (WT or L858R) with and without coexpression of RUSH-EGFR in HEK293T cells. When expressed alone, HA-EGFR^WT^-FLAG exhibited typical cell surface localization, whereas HA-EGFR^L858R^-FLAG showed both surface and punctate intracellular distribution (Fig. 5, *Z*–*AA*, *AD*–*AE*). In coexpression experiments, we observed that RUSH-EGFR^WT^ remained ER localized, whereas HA-EGFR^WT^-FLAG reached the plasma membrane in all cells, consistent with monomeric behavior of WT EGFR in the ER (Fig. 5, *AB*–*AC*). Strikingly, coexpression of HA-EGFR^L858R^-FLAG with RUSH-EGFR^L858R^ resulted in ER retention of both constructs in all cells (Fig. 5, *AF*–*AG*). This colocalization strongly suggests that the L858R mutant forms dimers in the ER, which are subsequently retained. These findings indicate that the reduced activation of ER-retained EGFR^L858R^ is not because of impaired dimerization but rather to its failure to reach the plasma membrane where full activation can occur.

### The EGFR polyR motif and the D198 residue of SAR1A are not essential for the export of EGFR^L858R^ from the ER

**Figure 6:**
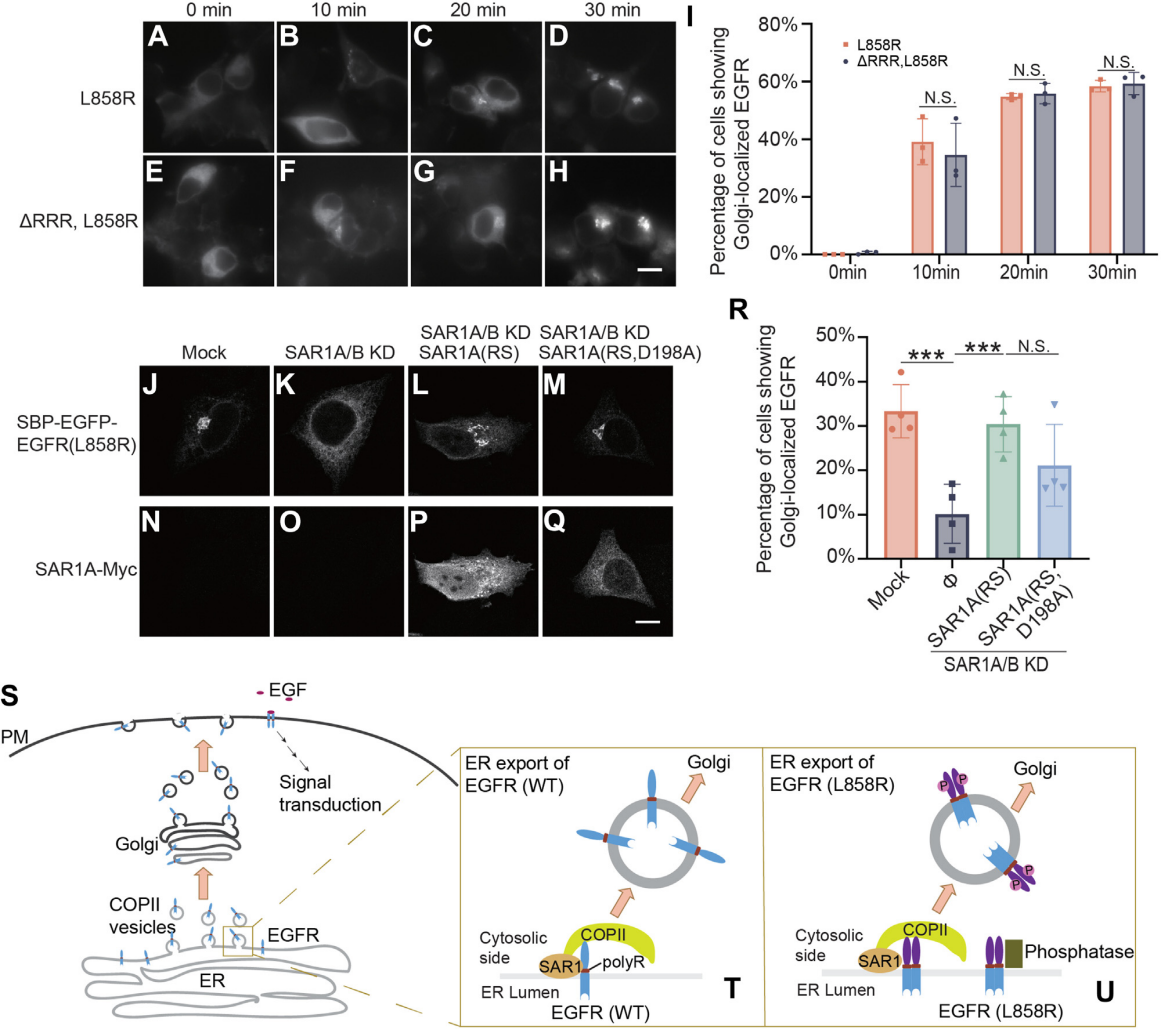
**EGFR polyR motif and the D198 residue on SAR1 are not essential for ER export of EGFR^L858R^**. *A*–*H*, the RUSH transport assay was performed in HEK293T cells transfected with the indicated RUSH constructs of EGFR. On day 1 after transfection, cells were incubated with biotin and cycloheximide for the indicated time. The localizations of the EGFR constructs were analyzed by immunofluorescence. Scale bar represents 10 μm. *I*, the percentage of cells showing Golgi-localized EGFR was quantified (n = 3, mean ± SD, >100 cells were quantified in each experimental group). NS, not significant. *J*–*Q*, the RUSH transport assay was performed in HeLa cells transfected with control siRNA or cotransfected with siRNAs against SAR1A and SAR1B. On day 1 after knockdown, cells were transfected with plasmids encoding Str-KDEL_SBP–EGFP–EGFR^L858R^ and the indicated SAR1A constructs. On day 2 after knockdown, cells were incubated with biotin and cycloheximide for 15 min. The localizations of EGFR^L858R^ were then analyzed. Scale bar represents 10 μm. *R*, the percentage of cells showing Golgi-localized EGFR^L858R^ was quantified (n = 3, mean ± SD, >100 cells were quantified in each experimental group). ∗∗∗*p* < 0.001; NS, not significant. *S*, our proposed model describing the molecular mechanisms regulating the export of EGFR^WT^ (*T*) or EGFR^L858R^ (*U*) from the ER. EGFP, enhanced GFP; EGFR, epidermal growth factor receptor; ER, endoplasmic reticulum; HEK293T, human embryonic kidney 293T cell line; PM, plasma membrane; polyR, polyarginine motif; RUSH, Retention Using Selective Hook.

Next, we investigated whether the ER export of EGFR^WT^ utilizes the same trafficking mechanism as EGFR^L858R^. SBP–EGFR–EGFR^L858R^ was efficiently delivered from the ER to the Golgi in a biotin-dependent manner (Fig. 6, *A*–*D*, *I*). Interestingly, deletion of the polyR motif on EGFR^L858R^, EGFR^ΔRRR, L858R^, did not show defects in ER-to-Golgi trafficking (Fig. 6, *E*–*H*, *I*). Double knockdown of SAR1A and SAR1B significantly reduced the kinetics of ER-to-Golgi trafficking of SBP–EGFP–EGFR^L858R^ (Fig. 6, *J*, *K* and *R*), and this defect was rescued by coexpression of siRNA-resistant SAR1A (Fig. 6, *L*, *P* and *R*), suggesting that SAR1A/B is essential for ER export of EGFR^L858R^. No significant difference was found in ER export of EGFR^L858R^ with SAR1A^RS^ and SAR1A^RS, D198A^ rescue in SAR1A/B knockdown cells (Fig. 6, *L*–*M*, *P*–*Q* and *R*), suggesting that the D198 residue is not essential for ER export of EGFR^L858R^. These results indicate that blocking the interaction between the polyR motif and SAR1A did not block ER export of EGFR^L858R^.

## Discussion

In this study, we demonstrate that the newly synthesized EGFR on the ER membrane is recognized by the SAR1 subunit of the COPII coat through the direct binding of the polyR motif and the D198 residue on SAR1. This interaction enables the sorting and concentration of EGFR in COPII-coated vesicles, ultimately leading to its export out of the ER en route to the Golgi (Fig. 6, *S* and *T*). The D198 residue of SAR1 was also shown to directly interact with the C-terminal (R/K)x(R/K) motif of GGTs to enrich GGTs at the ER exit sites (9). In addition, it was found that the E62,63 residues on SAR1 recognize polybasic motifs on Frizzled-6, facilitating the ER export of Frizzled-6. Interestingly, the D198A mutation in SAR1 showed no defects in its interaction with the polybasic motif of Frizzled-6 (4). These findings collectively underscore the crucial role of SAR1 in cargo selection, demonstrating its ability to utilize distinct acidic motifs to recognize polybasic motifs on various cargo proteins.

It has been reported that knocking down the specific isoform SEC24D in HeLa cells significantly impaired the transport efficiency of EGFR from the ER, suggesting that EGFR is a cargo client of SEC24D in COPII trafficking (2). However, it remains unclear whether SEC24D directly binds to EGFR. Our results showed that SAR1 directly interacts with EGFR to facilitate cargo recognition. These analyses suggest that the ER export of EGFR might depend on multiple factors to ensure both specificity and efficiency. Further research is needed to clarify the exact role of SEC24D in this process and to explore other factors involved in EGFR trafficking.

The L858R mutation in exon 21 of EGFR is a prevalent mutation in cancer cells. Structural analyses have shown that residue L858 is crucial for hydrophobic interactions within the EGFR kinase domain that stabilize its inactive conformation. Substituting L858 with arginine introduces a larger, charged side chain that disrupts these interactions and results in a constitutively active kinase state (5). Our findings suggest that, unlike WT EGFR, the polyR motif is not essential for the surface delivery of the L858R mutant (EGFR^L858R^). A possible reason is that the conformational change in EGFR^L858R^ may create steric hindrance, preventing the polyR motif from effectively binding with the D198 residue of SAR1. We hypothesize that while the ER export of EGFR^L858R^ relies on the conventional COPII-mediated pathway, it may require mechanisms other than the direct interaction between the polyR motif and the D198 residue of SAR1.

Intriguingly, our study revealed that the constitutively active L858R mutant remains unphosphorylated within the ER and only becomes phosphorylated after its release. This might be attributed to the activity of ER-resident phosphatases such as protein tyrosine phosphatase 1B and protein phosphatase 2Ce, which potentially dephosphorylate the active EGFR within the ER to keep signaling subdued under normal conditions (12, 13) (Fig. 6, *U*). This aspect underscores a complex regulatory mechanism that manages kinase activity and trafficking within the cell.

EGFR is a critical target for anticancer therapies. Our studies show that the ER export of both WT EGFR and the oncogenic L858R mutant is crucial for their activation. This suggests that inhibiting the ER export of EGFR could represent a novel strategy for downregulating its activity in cancer treatment. Our experiments demonstrate that a peptide containing the polyR motif of EGFR significantly reduces the packaging efficiency of EGFR^WT^ into COPII vesicles *in vitro*. This indicates that the polyR peptide, or an analog, could potentially serve as a therapeutic agent in EGFR-related cancers. Further research into the surface delivery mechanisms of the EGFR^L858R^ mutant and the development of drugs targeting this form of EGFR offers a promising direction for creating more effective treatments for L858R-specific cancers.

## Experimental procedures

### Constructs, reagents, cell culture, and transfection

The plasmid encoding Str-KDEL_SBP–EGFP–EGFR was generated as described previously (10). The plasmids encoding Str-KDEL_SBP–EGFP–EGFR^ΔLD^, Str-KDEL_SBP–EGFP–EGFR^Δcyto^, and Str-KDEL_SBP–EGFP–EGFR^ΔcytoRRR^ were generated to encode EGFR fragments spanning amino acids 646 to 1210, 31 to 668, and 31 to 671, respectively. The plasmid encoding Str-KDEL_SBP–EGFP–EGFR^ΔcytoAAA^ was generated to encode EGFR fragment spanning amino acids 31 to 668 followed by three alanine residues. The plasmids encoding Str-KDEL_SBP–EGFP–EGFR^ΔRRR^, Str-KDEL_SBP–EGFP–EGFR^L858R^, and Str-KDEL_SBP–EGFP–EGFR^ΔRRR,L858R^ were generated by QuikChange II site-directed mutagenesis using PfuUltra High-Fidelity DNA polymerase (Agilent Technologies). The plasmid encoding EGFR-GFP was generated by inserting DNA fragment encoding human EGFR into pEGFP-N1 vector. The plasmid encoding EGFR(ΔRRR)-GFP was generated by QuikChange II site-directed mutagenesis. The plasmid encoding EGFR-HA was generated by cloning full-length EGFR amplified from EGFR-GFP into pcDNA3.1 vector, and 3xHA tag was inserted by PCR to the C terminus of EGFR. The plasmids encoding GST-tagged human SAR1A and His-tagged human SAR1A were generated as described previously (14). The plasmid encoding His-tagged SAR1A(Δ2-17, H79G) was generated as described previously (8). The plasmid encoding SAR1(rescue)-Myc was ordered from Beijing Genomics Institute. The D198A mutations in GST-SAR1(D198A), SAR1(Δ2-17,H79G,D198A)-His, SAR1(rescue,D198A)-Myc, and SAR1(D198A)-DsRed were made by QuikChange II site-directed mutagenesis.

siRNAs against SAR1A and SAR1B were purchased from Ruibo Bio. The target sequence against SAR1A is GGAATGACCTTTACAACTT. The target sequence against SAR1B is CTGGTAAACTGGTATTTCT. The commercial antibodies were sheep anti-TGN46 (AbD Serotec; catalog number: AHP500G), mouse anti-Myc (Cell Signaling; catalog number: 2276), mouse anti-actin (Proteintech; catalog number: 60008-1), rabbit anti-phospho-EGFR (Cell Signaling; catalog number: 3777), mouse anti-EGFR (Santa Cruz; catalog number: sc-101), and rabbit anti-HA (Cell Signaling; catalog number: 3724). Rabbit anti-SAR1A/B, rabbit anti-ERGIC53, rabbit anti-SEC22B, and rabbit anti-SEC23A were kindly provided by Prof Randy Schekman (University of California, Berkeley). Rabbit anti-GFP was a gift from Prof Robert Qi (The Hong Kong University of Science and Technology). The specificity of the antibodies was validated by our previous publications. Peptides used in the *in vitro* vesicle formation assays and peptide-binding assay were purchased from GenScript.

The HeLa and HEK293T cell lines were generously supplied by the University of California-Berkeley Cell Culture Facility, with their identity verified by short tandem repeat profiling. The COS-7 cells were acquired from American Type Culture Collection (catalog no.: CRL-1651; Research Resource Identifier: CVCL_0224). HeLa, HEK293T, and COS7 cells were maintained in GIBCO Dulbecco’s modified Eagle’s medium containing 10% fetal bovine serum, 10 milliunits/ml penicillin, and 0.1 mg/ml streptomycin. Transfection of siRNA or DNA constructs into HeLa cells, HEK293T cells, or COS7 cells and immunofluorescence were performed as described previously (14, 15). Images were acquired with a Zeiss Axio Observer Z1 microscope system (Carl Zeiss) equipped with an ORCA Flash 4.0 camera (Hamamatsu) or Leica TCS SP8 Confocal Laser Scanning Microscope (Leica).

### RUSH assays, cell treatments, immunofluorescence, and cell lysate preparation

RUSH assays were performed by treating HeLa or HEK293T cells transfected with plasmids encoding Str-KDEL and different versions of SBP–EGFP–EGFR in complete medium containing 40 μM biotin (Sigma–Aldrich) and 100 ng/μl cycloheximide (Sigma–Aldrich) for the indicated time. Cells were fixed by 4% paraformaldehyde mounted on glass slides by ProLong Gold Antifade Mountant with 4′,6-diamidino-2-phenylindole (Invitrogen) for microscope analysis. For EGF treatment, HeLa cells were incubated with 10 ng/ml EGF (Sigma–Aldrich) at 37 °C overnight (around 20 h).

Immunofluorescence was performed as described (14). Cells were fixed by 4% paraformaldehyde in PBS for 20 min. After fixation, cells were incubated with blocking buffer (2.5% fetal bovine serum, 0.1% Triton X-100, and 0.2 M glycine in PBS) for 30 min at room temperature. Cells were then incubated with indicated primary antibody in blocking buffer at room temperature for 30 min and washed three times with PBS, followed by incubation with secondary antibodies in dark for 30 min in blocking buffer at room temperature. Cells were washed three times with PBS before being mounted onto slides with ProLong Gold Antifade Mountant with 4′,6-diamidino-2-phenylindole for microscope analysis.

To prepare cell lysates for detecting phosphorylated EGFR, cells were first washed and harvest in ice-cold KOAc buffer (110 mM KOAc, 20 mM Hepes, 2 mM MgOAc, pH 7.2) and then centrifuged at 300g for 3 min. The cell pellets were resuspended in protein sample buffer (88 mM KOAc, 16 mM Hepes, 1.6 mM MgOAc, 0.4% Triton X-100, 0.2% bromophenol blue, 0.8% SDS, 4% glycerol, and 5% β-mercaptoethanol), and mixed thoroughly by passing through a 25G needle attached to a 1 ml syringe 100 times. The mixture was incubated at 100 °C for 10 min and analyzed by immunoblotting.

### *In vitro* vesicle formation assay

*In vitro* vesicle formation assay was performed as described (14). HEK293T cells transfected with Str-KDEL_SBP–EGFP–EGFR or Str-KDEL_SBP–EGFP–EGFR^ΔRRR^ were harvested and permeabilized in ice-cold KOAc buffer containing 40 μg/ml digitonin on ice for 5 min. The permeabilized cells were collected by centrifugation at 300*g* for 3 min at 4 °C, washed with KOAc buffer, and resuspended in KOAc buffer. The semi-intact cells were then incubated at 32 °C with 2 mg/ml rat liver cytosol, 200 μM GTP (Wako; catalog number: SAH3766), 40 μM biotin (Sigma–Aldrich), and an ATP regeneration system (4 mM creatine phosphate [Roche], 0.02 mg/ml of creatine phosphokinase [Roche], and 100 μM ATP [Sigma]) in the presence or the absence of 0.01 μg/μl SAR1A (H79G) mutant protein. After a 1 h incubation, the reaction mixture was centrifuged at 14,000*g* at 4 °C for 20 min to remove the ER and Golgi membranes, nucleus, as well as other cell debris from the reaction mixture. The supernatant fraction containing the released vesicles was resuspended in 35% Opti-Prep and overlaid with 30% Opti-Prep. KOAc buffer (60 μl) was added on the top of the step gradient of Opti-Prep. The Opti-Prep gradients were then centrifuged at 100,000*g* in a TLS55 (Beckman) or S55S (Hitachi) rotor at 4 °C for 1.5 h. After centrifugation, the top 200 μl fraction was collected and centrifuged at 100,000*g* in a TLA120.1 (Beckman) or S120AT3 (Hitachi) rotor at 4 °C for 30 min to sediment vesicles. The vesicle pellet was resuspended in KOAc buffer and analyzed by immunoblot. Data were collected by ChemiDoc imaging system and analyzed by ImageJ. Quantification was performed by Student's *t* test under two-tailed and unpaired condition.

### Protein purification, GST pull-down assay, and peptide-binding assay

Purification of GST-tagged SAR1A and His-tagged SAR1A was performed as described previously (14, 15). For GST-pull down assay, COS7 cells were transfected with EGFR–EGFP or EGFR-HA and harvested and incubated with purified GST protein in the addition of GDP or GTPγS for 3 h in 4 °C with rotation. After incubation, beads were washed for three times with wash buffer (110 mM potassium acetate, 20 mM Hepes, pH.7.2, 2 mM magnesium acetate, and 0.5% Triton X-100) and incubated with 2X protein sample buffer (100 mM Tris–HCl [pH 6.8], 0.2% bromophenol blue, 4% SDS, 20% glycerol, and 25% β-mercaptoethanol) at 55 °C for 30 min for Western blotting analysis and Ponceau S staining (0.5% [w/v] Ponceau S, 1% acetic acid).

For peptide-binding assay, synthetic polyR peptides (RRRHIVRKRTLRRC) were purchased from GenScript and coupled to thiopyridone-Sepharose 6B beads (Sigma–Aldrich) *via* the added C-terminal cysteine residue. The coupling reaction was performed based on previous reports (16, 17). For binding experiments, 2 μg purified His-tagged SAR1A was preincubated at 4 °C for 30 min with 500 μM GTPγS in a total volume of 15 μl HK buffer (100 mM KCl, 20 mM Hepes, pH 7.2). After incubation, 15 μl buffer containing around 5 μl beads with 5 nmol of peptides was added to the reaction mixture for 1 h at 4 °C. The beads were washed four times with 500 μl of HK buffer and analyzed by immunoblotting.

## Data availability

All data are contained within the article and accompanying supporting information.

## Conflict of interest

The authors declare that they have no conflicts of interest with the contents of this article.
